# A Young Male With an Active Giant Cell Tumor: A Case Report

**DOI:** 10.7759/cureus.30389

**Published:** 2022-10-17

**Authors:** Avantika Thakur, Sanjay V Deshpande

**Affiliations:** 1 Orthopaedic Surgery, Jawaharlal Nehru Medical College, Datta Meghe Institute of Medical Science, Wardha, IND; 2 Orthopaedics and Traumatology, Jawaharlal Nehru Medical College, Datta Meghe Institute of Medical Science, Wardha, IND

**Keywords:** trauma, surgery, recurrence, bone grafting, giant cell tumor

## Abstract

A giant cell tumor (GCT) is a benign but locally aggressive tumor usually present over the knee joint. Its etiology is unknown but some studies have shown that it appears due to overexpression in RANK/RANKL by neoplastic mononuclear stromal cells signaling pathway, which results in hyperproliferation of osteoclasts. This is a case of a 25-year-old male who presented with swelling associated with pain over his left distal femur since eight months. The range of motion (ROM) at the knee joint was painful from 0 to 110 degrees and no knee effusion was observed. Examination revealed a slightly mobile mass present over the knee joint. Additional preoperative workup such as Computed Tomography (CT) scan and Magnetic Resonance Imaging (MRI) was done. Histopathological findings showed the presence of large multinucleated osteoclast-like giant cells. Radiographs showed a permeative lytic lesion within the distal femur with surrounding cortical destruction. Surgical excision by curettage and bone grafting was done. The patient did well, without clinical recurrence at one-year follow-up. A local recurrence rate of 2.5 to 45% is observed. Aggressive operative excision, use of adjuvants at the time of resection, and ongoing postoperative monitoring can decrease patient morbidity.

## Introduction

A giant cell tumor (GCT) is a benign but locally aggressive tumor. It usually develops over the epiphysis near the joint. It is usually located over the cortex of the epiphysis of a long bone. The periarticular area of the knee joint is the most common area involved, though it can occur throughout the body including in the upper extremity and axial skeleton. It usually has a size of 2 to 4 cm and can have soft tissue extension but does not arise in the soft tissues and is 5 cm if present in deep tissues [1-4]. It is commonly seen in young adults aged 20-40. Especially those aged 20-30 are more prone to this disease [5]. Etiologically, this disease occurs due to overexpression in RANK/RANKL by neoplastic mononuclear stromal cells signaling pathway, which results in hyperproliferation of osteoclast but can sometimes be associated with Paget disease of bone [6]. The prevalence of GCT is higher in females, with a ratio of 1.38:1 [5]. It usually occurs in the femur and has a high prevalence of 35%, ahead of the tibia at 18% [6]. Histological findings show the presence of large multinucleated osteoclast-like giant cells and mononuclear spindles like stromal cells and other monocytes. The workup of a patient with a suspected lesion should involve blood work as few parameters can detect relapse or degree of osteolysis, but the main diagnosis is by biopsy and radiology. A radionuclide bone scan is done to detect the disease and tumor. Molecular findings show cells with H3.3p.Gly34 mutation [6]. Treatment is usually done surgically as the tumor is excised. Amputation, bone grafting, and bone reconstruction are also used as surgical options for treating patients [7]. Radiation therapy is mainly used in recurrence, as palliative or in cases that cannot be operated on due to other comorbidities [8]. The complication of GCT could be pathologic fracture, recurrence, pulmonary metastases, or nerve root compression in the vertebral column. After surgery, the patient is sent for rehabilitation after one week of bed rest to reconstruct the bone [6]. Recurrence of aggressive tumor-like GCT is normal even after excision with a rate of 2.5 to 45% depending on the type of surgical procedure and local presentation of the tumor.

## Case presentation

A 25-year-old male patient came to the hospital with chief complaints of pain and swelling simultaneously in the left knee since eight months. The patient gave an alleged history of slip and fall while playing (eight months back), after which he developed pain and swelling in his left knee, which was sudden in onset, constant throughout so much that he used to limp while walking with ROM 0 to 110 degrees but painful. After two months, the pain subsided, and it was easier for him to walk, but since the last two months, he again felt pain and swelling along with the instability of his left knee while walking. The patient had no significant associated illness. On general examination, nothing abnormal was found. On local examination, diffuse swelling over the left distal femur was inspected. The overlying skin was also tensed. On palpation, a local temperature rise was observed over the lateral aspect of the left distal femur and knee. The patellar tap was negative.

**Figure 1:**
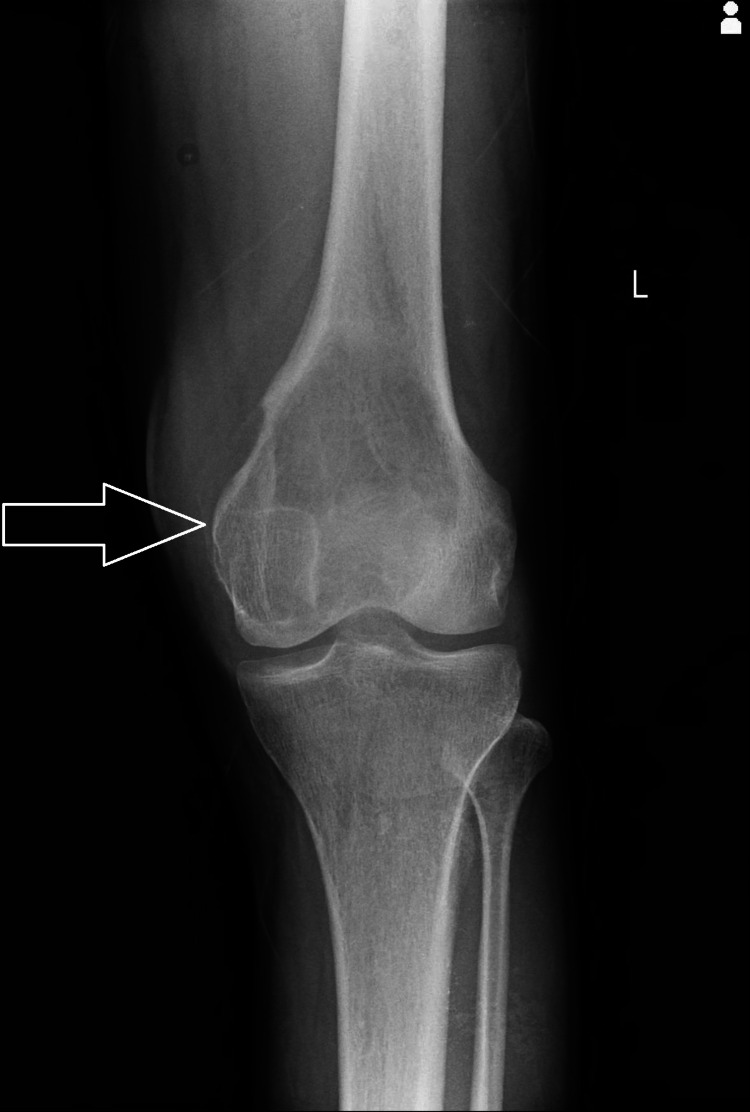
X-ray having homogenous radio-opaque area with a lytic appearance involving epiphysis of distal femur - anteroposterior view

**Figure 2:**
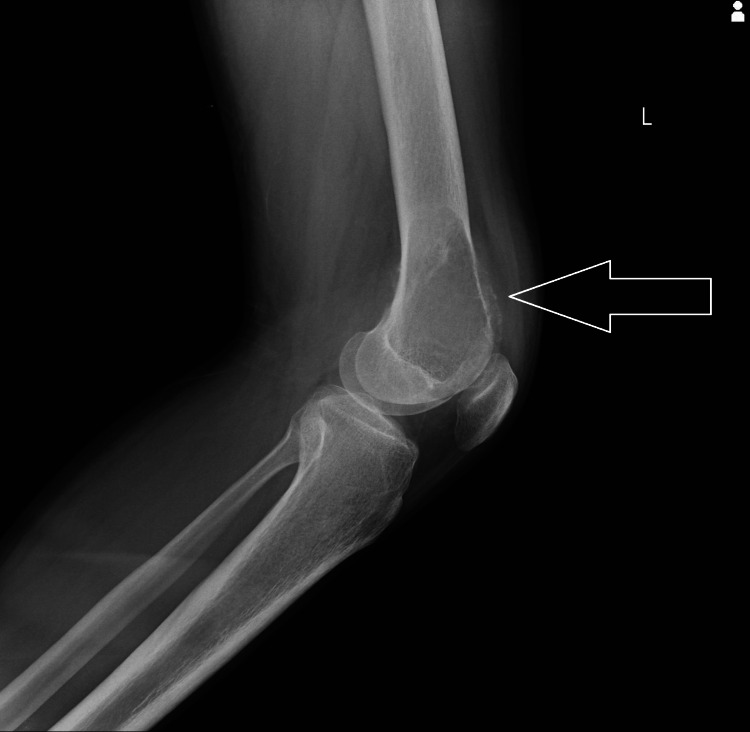
X-ray showing homogenous radio-opaque area with lytic appearance involving epiphysis of distal femur - lateral view

The provisional diagnosis was suggestive of an aneurysmal bone cyst left distal femur. The patient was advised to have a blood examination, but no such finding was observed. On x-ray, a homogenous radio-opaque area with a lytic appearance involving the epiphysis and extending into the metaphysis (Figures 1, 2).

MRI showed well-defined expansile multiloculated lesions in the distal metaphysis of the femur with multiple fluid levels. Biopsy of a tumor, which was multiple, irregular, yellowish brown tissue, was microscopically examined and confirmed GCT. Biopsy of the synovial membrane showed fibro adipose muscular tissue with congested blood vessels, and scattered chronic non-specific inflammatory infiltrate. A biopsy of the medial wall showed osseocartilaginous tissue with a tumor rim showing malignant spindle cells and giant cells. Biopsy results of the periosteum showed unremarkable osseocartilaginous tissue with fibro adipose muscular tissue, congested blood vessels, and stromal tissue element with minimal chronic non-specific inflammation.

**Figure 3:**
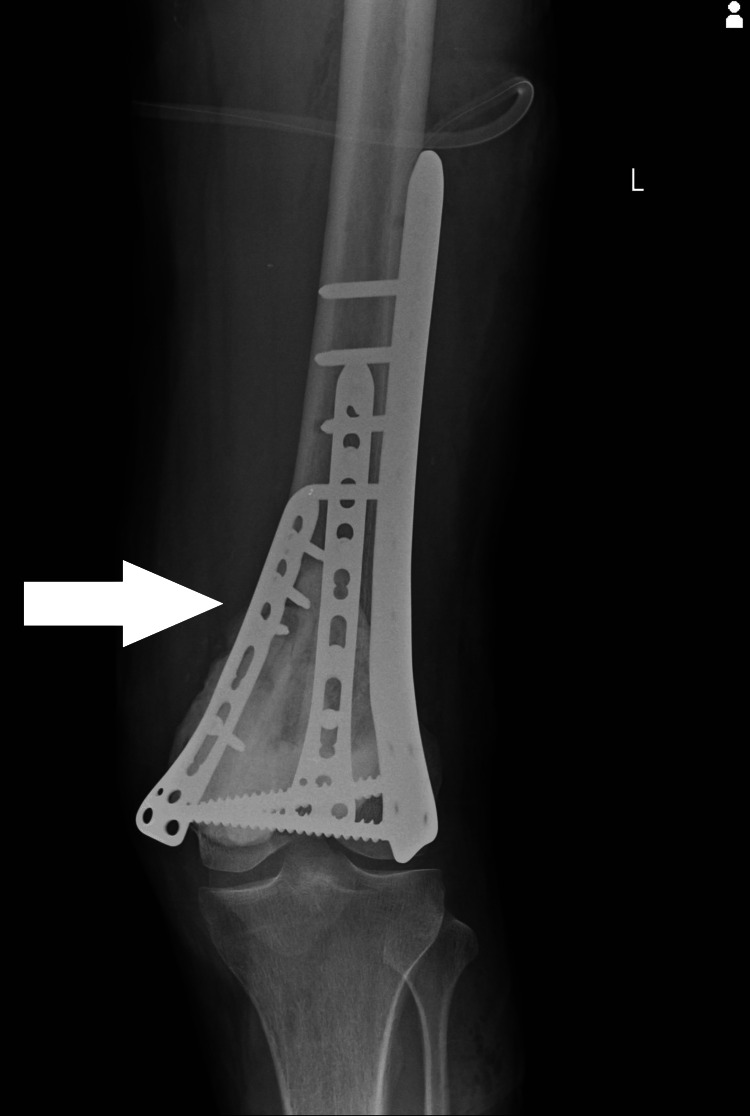
X-ray image depicting locking plate used for bone grafting

**Figure 4:**
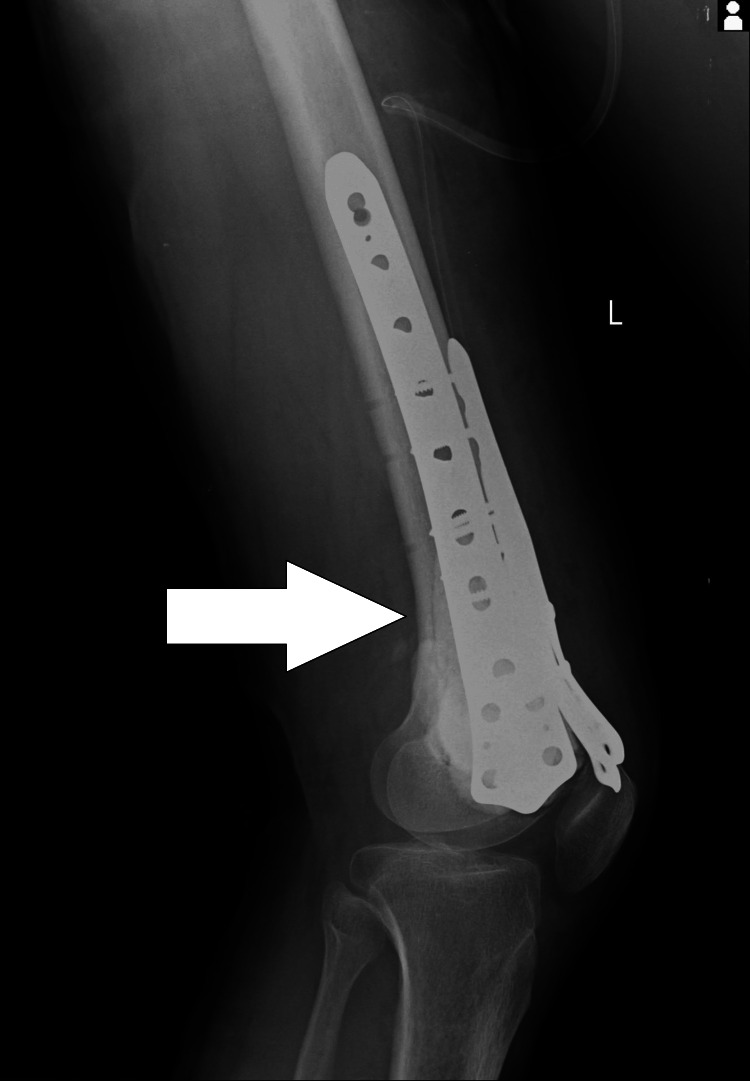
X-ray image showing lateral view post operative

By using the surgical approach of curettage the periosteum was removed involved with the tumor. Anterior and medial cortex was involved within the tumor, and the cortex was broken. Curettage and excision of the complete tumor were done. Approximately 8 cm fibula was cut with the help of a bone saw. A seven-hole lateral distal femur locking plate was used and fixed over the lateral aspect of the femur with three proximal and two distal screws. The fibula graft was cut into two pieces. One piece of the graft that was 4 cm was fixed with the distal femur medial contoured three-hole locking plate and fixed on the medial aspect of the distal femur with two screws. A 4 cm piece of fibula graft was fixed with a six-hole anteromedial locking contoured distal femur plate and fixed with two screws over the anterior aspect of the distal femur (Figures 3, 4). Bone cement polymethyl methacrylate (PMMA) 70 grams was used to fill up the void in the distal femur. Anteromedial and anterior cortex distal femur were thus reconstructed.

Twelve stitches were removed two weeks after operation. The patient was suggested for physiotherapy for at least two months postoperative. The patient was given a time of 24 months of follow-up. Current follow-up shows no trace of recurrence of GCT. Radiographic monitoring for recurrence is done in this patient.

## Discussion

Usually, GCT is of two types: conventional giant cell tumor of bone and malignant giant cell tumor of bone. According to our case, the patient had a conventional giant cell tumor of bone as he had features like that of a conventional giant cell tumor [6]. Features were it was eccentric and lacked peripheral bone sclerosis.

Radioimaging and histology are very crucial investigations for diagnosing tumor cases. X-ray usually shows osteolytic or radiolucent lesions and well-defined non-sclerotic margins and eccentric location. The plain radiograph shows narrow zones of transition, cortical thinning, and expansile remodeling. MRI finding shows hemorrhagic areas and fluid-fluid levels, suggesting aneurysmal bone cyst-like changes [6]. Macroscopically, they are variable in appearance with soft friable red tarnish hemorrhagic areas of blood-filled cystic spaces admixed with cream-yellow and firm whitish-colored areas corresponding to xanthomatous changes and fibrous tissue. The microscopic view shows multiple osteoclastic giant cells with neoplastic mononuclear cells in between. Haemorrhagic, aneurysmatic changes and foamy macrophages can be observed. Cortical bone erosion with new bone formation in the periphery of the tumor is characteristically observed with confirmatory pathological testing for 20q11 amplification [6]. Differential diagnoses with such features can get confused with giant cell reparative granuloma, brown tumor, osteoblastoma, chondroblastoma, non-ossifying fibroma, and osteosarcoma. So with the help of radioimaging, a final diagnosis can be made.

Surgical treatment includes amputation, bone grafting, reconstruction, removal of the tumor and damaged bone, and radiation in which the tumor cannot be excised. In our case, bone grafting and reconstruction were done after the removal of the tumor. It is considered the safe method as the tumor had already eroded some part of the cortex of the femur. Part of the fibula bone was grafted over the excised part of the femur. Amputation is typically reserved for non-reconstructable areas or recurrences and hence was not required in this patient. Advantages of bone grafting are it undergoes remodeling along stress lines, and once incorporated, reconstruction is permanent [6].

Denosumab is considered an effective treatment against GCT as tumor regression is observed [8,9]. The drug is commonly used to shrink and reduce the tumor volume and postoperative help in increasing bone mass by reducing bone resorption.

## Conclusions

GCT is not only benign but could be malignant. GCT is an aggressive tumor more prevalent in females but males can also suffer from such disease. The etiopathogenesis of the disease is still unknown. Radio imaging and histopathology together play a crucial role in diagnosing the tumor. Since the tumor is aggressive, recurrence chances of the tumor even after surgical excision is possible. In some cases when excision is not possible chemotherapy is the best choice. Proper care after surgery needs to be taken. Physiotherapy of the affected limb after surgery should be done.
